# Supplementary material for: Topological Resistance-Free One-Way Transport in a Square-Hexagon Lattice Gyromagnetic Photonic Crystal
Source: Nanomaterials (Basel). 2022 Aug 30;12(17):3009. doi: 10.3390/nano12173009 (PMC9458195; doi:10.3390/nano12173009)
Supplement: Supplementary file 1 [file nanomaterials-12-03009-s001.zip › nanomaterials-1859986-supplementary.pdf]

nanomaterials

Article

Topological Resistance-Free One-Way Transport in a Square-Hexagon Lattice Gyromagnetic Photonic Crystal

Qiumeng Qin [1,†], Jianfeng Chen [1,*,†], Hao Lin [1], Chaoqun Peng [1] and Zhi-Yuan Li [1,2,*]

1 School of Physics and Optoelectronics, South China University of Technology, Guangzhou 510640, China;
2 State Key Laboratory of Luminescent Materials and Devices, South China University of Technology, Guangzhou 510640, China
* Correspondence: author: phjfchen@mail.scut.edu.cn (J.C.); phzyli@scut.edu.cn (Z.-Y.L.)
† These authors contributed equally to this work.

I. Structural and material properties

The photonic crystal of YIG cylinders is placed in an air-loaded planar waveguide sandwiched between two parallel aluminum alloy layers that are used to forbid the electromagnetic (EM) waves from leaking off in the z direction. The relative permittivity and permeability of air are ε_1=1 and μ_1=1, respectively. The YIG material has a measured relative permittivity 14.5, a dielectric loss tangent 0.0001, a measured saturation magnetization M_s=1950 Gauss and a ferromagnetic resonance linewidth of 20 Oe. Typically, the magnetization will decrease to a negligible value without the presentence of the external magnetic field. The relative magnetic permeability of YIG has the form

$$\hat{\mu}=\begin{pmatrix} \mu_r & i\mu_k & 0 \\ -i\mu_k & \mu_r & 0 \\ 0 & 0 & 1 \end{pmatrix}, \qquad \text{(S1)}$$

where $\mu_r = 1+\frac{(\omega_0+i\alpha\omega)\omega_m}{(\omega_0+i\alpha\omega)^2-\omega^2}$, $\mu_k = 1+\frac{\omega\omega_m}{(\omega_0+i\alpha\omega)^2-\omega^2}$, $\omega_m=\gamma M_s$, $\omega_0=\gamma H_0$, H_0=0.05 T is the external magnetic field, γ=1.76×10^{11} S^{-1}T^{-1} is the gyromagnetic ratio, α=0.0001i is the damping coefficient, and ω is the operating frequency.

II. Experimental measurement and numerical calculation methods

There are six layers in the entire experimental configuration, with the top (1st) being the metal plate, the second and bottom (6th) layers being the square-hexagonal array of NdFeB permanent magnet rods, the fourth layer being the gyromagnetic photonic crystal structures made from square-hexagonal array of gyromagnetic cylinders, and the third and fifth layers being the metal confinement layers. Two aluminum alloy plates with thickness h_2=h_6=2.0 mm are set in the second and sixth layers. A square-hexagonal array of holes is drilled into both plates, and a pair of N and S NdFeB permanent magnet rods are pressed tightly into these face-to-face holes in these two layers, so that the displacement of magnets owing to the attraction and repulsion of different magnets is completely avoided. Each NdFeB cylinder is of radius 2.0 mm and height 2.0 mm. These NdFeB cylinder pairs can apply one-to-one external magnetic field to the gyromagnetic cylinders. The crystal of gyromagnetic cylinders (fourth layer with height h_4=5.0 mm) is placed in an air-loaded planar waveguide sandwiched between two parallel aluminum alloy layers (i.e. third and fifth layers with height h_3=h_5=1.0 mm) that are used to completely forbid the electromagnetic waves from leaking off in the z direction. The array is composed of 16×12 rotated square lattices along the x and y directions, and the lattice constant is a=14.0 mm and the radius of gyromagnetic cylinder is r=1.5 mm. The metallic strips are placed near the boundaries of gyromagnetic photonic crystal to form the transport channels, and the microwave absorbers are utilized to avoid the reflection of electromagnetic waves. The

two configurations are described by perfect electrical conductor boundary conditions and scattering boundary conditions in numerical simulations. In this case, the polarization is identical to the pure transverse electric (TE) states in two-dimensional photonic crystals. The transmission measurement is made with the Keysight P9374A vector network analyzer, which supports electromagnetic waves with frequency ranging from 300 kHz to 20 GHz. The feed and detect probes are inserted at each end of the boundary for measurement.

All the numerical simulations for gyromagnetic photonic crystals are calculated by using the commercial software COMSOL MULTIPHYSICS with RF module in frequency domain. In this case, the polarization is identical to the pure TE states (where electric field is parallel to z direction) in two-dimensional photonic crystals, so only TE polarization is considered.

As a typical example, the calculation details of Edge 1 can be seen in Figs. S1 and S2.

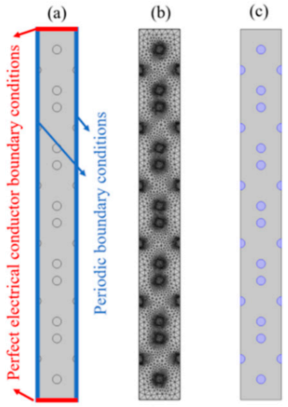

Figure S1. Schematic of the supercell of gyromagnetic photonic crystal of Edge 1. (a) Boundary conditions. The red and blue lines are set as the perfect electrical conductor boundary conditions and the periodic boundary conditions. (b) Mesh of supercell. The maximum and minimum dimensions of the mesh are 0.851cm and 0.004 cm respectively, which are much smaller than the excitation wavelength of about 1.689 cm (the excitation frequency is 17.76 GHz) to ensure the convergence of the calculated results. (c) The external magnetic field distributions of YIG crystals: the external magnetic field is along +z direction, marked in blue circle.

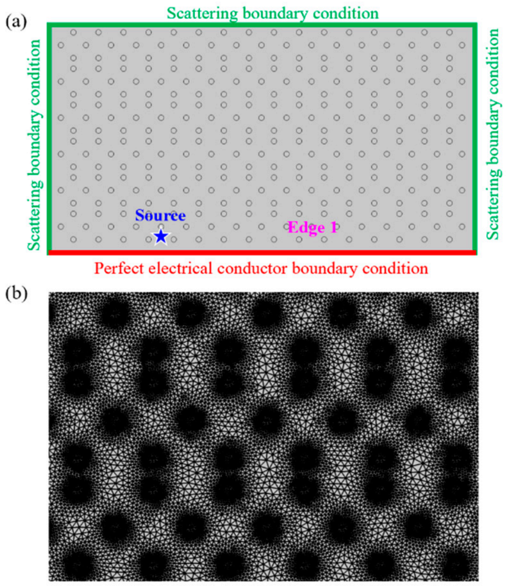

Figure S2. Simulated structure for the field distribution at Edge 1. (a) Boundary conditions and excitation. The red line is set as the perfect electrical conductor boundary condition, while the green lines are set as the scattering boundary conditions, and the blue star is the line source. (b)

Mesh of simulated structure. The maximum and minimum dimensions of the mesh are 1.300 cm and 0.007 cm respectively, which are much smaller than the excitation wavelength about 1.689 cm (the excitation frequency is 17.76 GHz) to ensure the convergence of the calculated results.

III. The perturbation of external magnetic field

The influence of magnetic field perturbation on the transport behavior of one-way edge states is very weak. To verify this point, as a typical example, we calculate the projected band structures of Edge 1 under the external magnetic field of 400 Gauss, 500 Gauss and 600 Gauss, as shown in Fig. S3. One can see that, at the work frequency of 17.45~17.95 GHz, the perturbation of external magnetic field strength has almost no influence on the transport properties of one-way edge states.

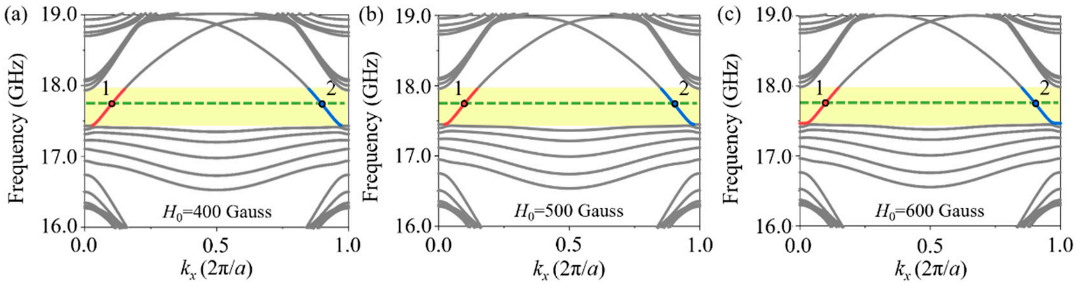

Figure S3. Projected band structures of Edge 1 under different external magnetic fields. (a) 400 Gauss. (b) 500 Gauss. (c) 600 Gauss.

IV. Topologically protected one-way edge states at various types of edges

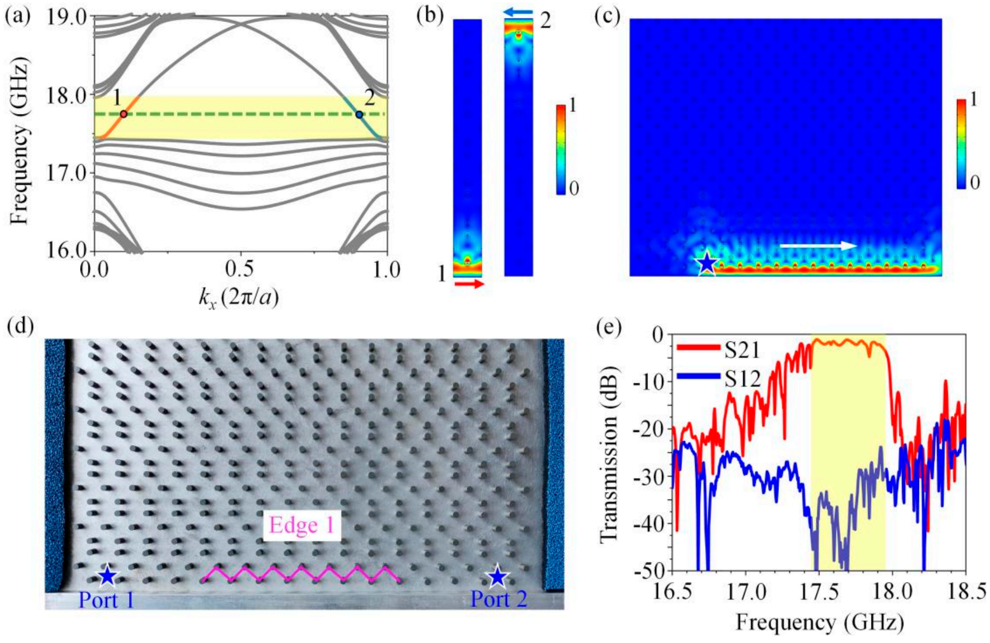

Figure S4. One-way edge states of Edge 1. (a) Projected band structures, showing two confined edge states within the photonic bandgap. The state 1 and state 2 are colored in red and blue, respectively. (b) Eigenmodal fields of state 1 and state 2 denoted in (a). The red and blue arrows indicate the right- and left-propagating edge states, respectively. (c) Simulated electric field distribution excited by a line source (denoted by the blue star) illustrating one-way transport of edge state. The white arrow represents the transport direction of edge state. (d) Photo picture of experimental sample. Two microwave absorbers are placed at the left and right boundaries of sample to avoid the reflection of EM waves, and a metallic strip is placed near the lower edge to form a transport channel. Edge 1 is highlighted in a magenta polyline at the lower edge. (e) Measured transmission spectra showing excellent one-way transport properties of edge states.

Edge 1. We first carry out an analysis on projected band structure, eigenmodal field and transmission spectrum of Edge 1. The outline of Edge 1 is marked with a magenta polyline at the lower edge, as illustrated in Fig. S4(d). One can see that it is a zigzag edge whose lattice constant along the x direction is a=14.0 mm. We perform a calculation to obtain the projected band structures. As displayed in Fig. S4(a), two dispersion curves (colored in red and blue respectively) appear within the bandgap (yellow region) ranging from 17.45 to 17.95 GHz. The eigenmodal fields of points 1 and 2 at a frequency of f_s=17.76 GHz are depicted in Fig. S4(b). There exist two edge states at the two parallel zigzag edges. As the slope signs of red and blue dispersion curves are positive and negative respectively, the lower and upper edge states will propagate rightwards (red arrow) and leftwards (blue arrow) respectively, showing apparent chirality. To examine the transport behaviors of these edge states, we build a realistic sample as illustrated in Fig. S4(d). When a line source (blue star) oscillating at f_s is placed at the lower edge, energy fluxes will be confined at the edge and unidirectionally propagate rightwards, as seen in Fig. S4(c). The measurements illustrated in Fig. S4(e) show that at the frequency range of 17.45~17.95 GHz (yellow region), the transmission parameter S21 (red curve) is much larger than S12 (blue curve) by about 35dB, so there exists a strong nonreciprocity between Port 1 and Port 2, in complete agreement with the simulation results of Figs. S4(a)-S4(c).

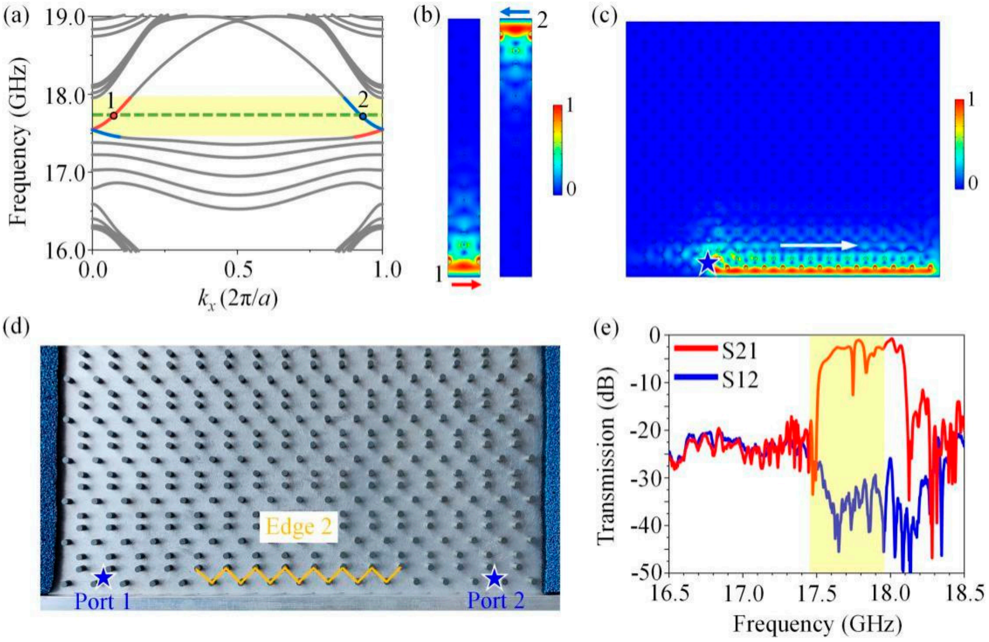

Figure S5. One-way edge states of Edge 2. (a) Projected band structures, showing two confined edge states within the photonic bandgap. (b) Eigenmodal fields of state 1 and state 2 denoted in (a). (c) Simulated electric field distribution excited by a line source illustrating one-way transport of edge state. (d) Photo picture of experimental sample. Edge 2 is highlighted in a yellow polyline at the lower edge. (e) Measured transmission spectra showing excellent one-way transport properties of edge states.

Edge 2. Next, we make an analysis over Edge 2. When the outermost row of YIG cylinders of Edge 1 is removed, the second type of edge (labeled as Edge 2) is obtained. The lattice constant of Edge 2 along the x direction is the same as that of Edge 1 (a=14.0 mm). The configuration of a realistic sample is illustrated in Fig. S5(d), where the shape of Edge 2 is outlined with a yellow polyline. The projected band structures depicted in Fig. S5(a) show that there exist two red and blue dispersion curves dwelling the bandgap (yellow region) with a frequency range of 17.45~17.95 GHz, and their slopes are opposite with each other. The eigenmodal field distributions of points 1 and 2 are calculated and illustrated in Fig. S5(b), and one can see that their electric fields are concentrated at the upper and lower edges respectively. Hence, there also exist two counter-propagating one-

way edge states at the two opposite edges, showing an obvious chirality. We place a line source oscillating at f_s to examine the electric field distributions. As seen in Fig. S5(c), a right-propagating one-way edge state is excited. We proceed to measure the transmission spectra in a realistic sample and find that the measured frequency range of one-way transport is basically consistent with the theoretical results, despite there exist some small frequency shifts, as plotted in Fig. S5(e).

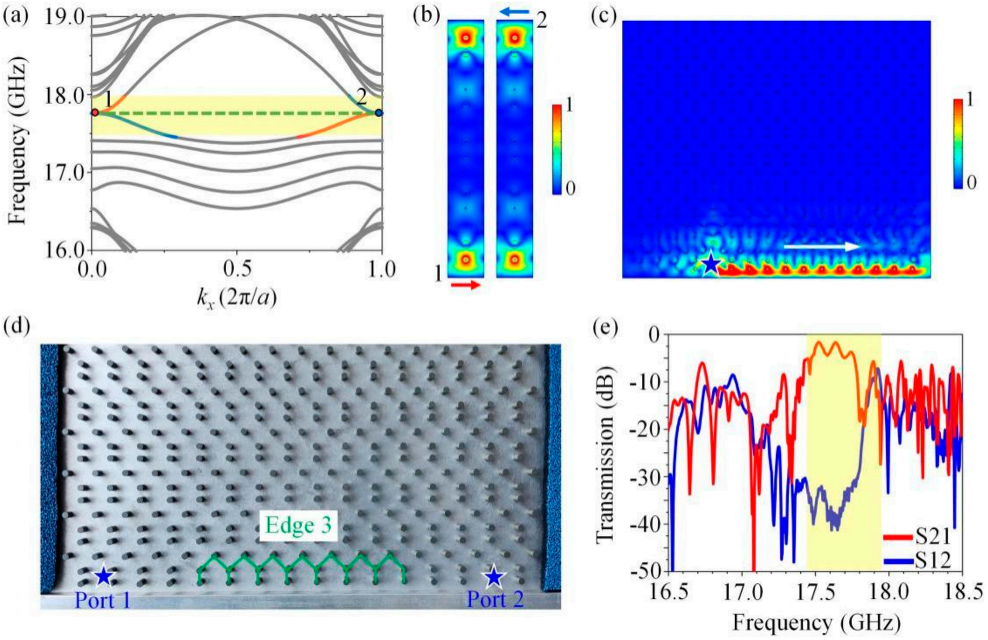

Figure S6. One-way edge states of Edge 3. (a) Projected band structures, showing two confined edge states within the photonic bandgap. (b) Eigenmodal fields of state 1 and state 2 denoted in (a). (c) Simulated electric field distribution excited by a line source illustrating one-way transport of edge state. (d) Photo picture of experimental sample. Edge 3 is highlighted in a green polyline at the lower edge. (e) Measured transmission spectra showing excellent one-way transport properties of edge states.

Edge 3. We turn on to make an analysis over Edge 3. When the outermost row of Edge 2 continues to be removed, Edge 3 (outlined with a green polyline) is formed, as seen in Fig. S6(d). We perform the numerical calculation of projected band diagram, eigenmodal field and electric field distribution, and the results are shown in Figs. S6(a)-S6(c). Fig. S6(a) shows that there still exist two opposite one-way dispersion curves residing within the bandgap ranging from 17.45 to 17.95 GHz (yellow region). However, in sharp contrast to the one-way dispersion curves of Edge 1 and Edge 2, two small exotic flat bands exist in both edges of the first Brillouin zone, indicating that the group velocities of the guided modes around the flat band are extremely low. When a line source with an excitation frequency of f_s is set on the lower edge, the radiating energy is strongly localized on the edge and transport rightwards with no backscattering, as depicted in Fig. S6(c). The one-way property of edge states is also reflected in the measured transmission spectra illustrated in Fig. S6(e), where the contrast of transmission parameters S21 and S12 at a frequency range of 17.45~17.95 GHz is about 35 dB.

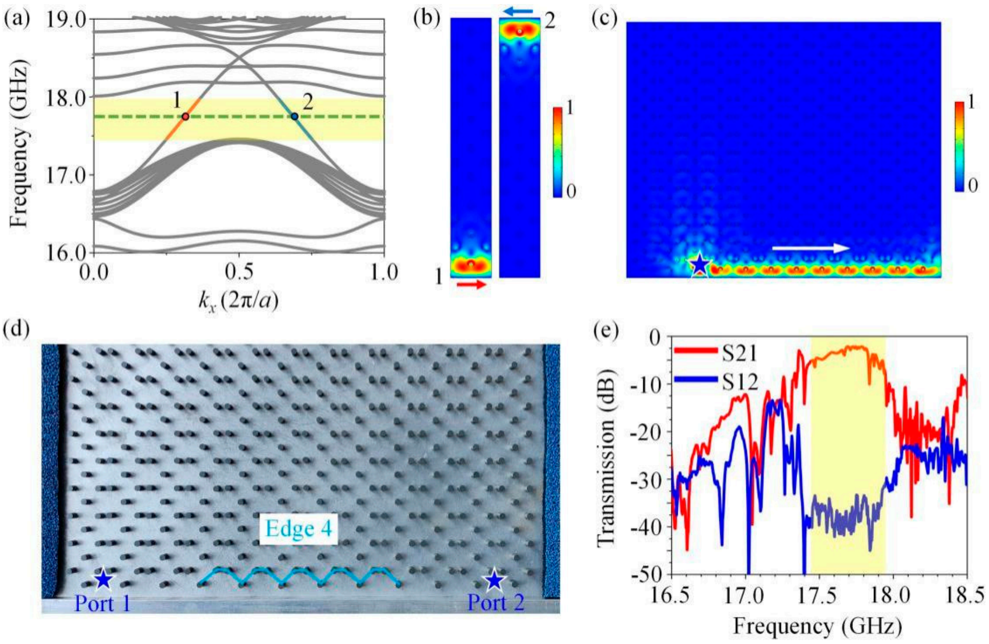

Figure S7. One-way edge states of Edge 4. (a) Projected band structures, showing two confined edge states within the photonic bandgap. (b) Eigenmodal fields of state 1 and state 2 denoted in (a). (c) Simulated electric field distribution excited by a line source illustrating one-way transport of edge state. (d) Photo picture of experimental sample. Edge 4 is highlighted in a blue polyline at the lower edge. (e) Measured transmission spectra showing excellent one-way transport properties of edge states.

Edge 4. Notice that the Edge 1-3 structures are all created at the zigzag edges along the x axis. Here, we turn our attention to the armchair edges along the y direction to investigate the transport behaviors of edge states. In these cases, the lattice constant is b=14$\sqrt{2}$mm. The projected band diagram, eigenmodal field and transport phenomenon of Edge 4 (outlined with blue polyline) are first calculated and discussed. Figs S7(a) and S7(b) show that there are two one-way edge states whose slopes are positive (state 1, colored in red) and negative (state 2, colored in blue) respectively in the bandgap ranging from 17.45 to 17.95 GHz (yellow region). In contrast to Edges 1-3 where the edge states are resided largely at both edges of Brillouin zone, the edge states of Edge 4 fall in the middle of Brillouin zone [$0.25<k_x<0.75$ ($2\pi/a$)]. Fig. S7(c) illustrates that when a line source (blue star) radiating at f_s is laid at the lower Edge 4, the electric fields are strongly concentrated on the first row of the gyromagnetic cylinders and propagates in only one direction (white arrow). The experimental sample and measured transmission spectra are plotted in Figs. S7(d) and S7(e) respectively. Obviously, in yellow region (17.45~17.95 GHz), the difference of S21 and S12 is about 35 dB, indicating that edge states exhibit the one-way transport property, in excellent agreement with the prediction made by projected band diagram in Fig. S7(a).

Edge 5. Finally, we make an analysis on Edge 5. When the outermost row of Edge 4 is taken away, Edge 5 emerges, marked as purple polyline emerges, as seen in Fig. S8(d). Fig. S8(a) shows that there are two one-way dispersion curves falling in the bandgap (yellow region). The eigenmodal fields of points 1 and 2 illustrated in Fig. S8(b) show that electric fields of eigenmodes 1 and 2 are strongly localized on the edges. So when a line source radiating at f_s=17.76 GHz is set on Edge 5, energy fluxes unidirectionally propagate to the right (white arrow), as seen in Fig. S8(c). The measured results plotted in Fig. S8(e) also indicate that between 17.45 to 17.95 GHz, the transmission coefficient S21 is much greater than S12, showing that EM wave propagates along the positive x direction and is prohibited along the negative x direction. More importantly, this frequency range of one-

way propagation is in good agreement with that of the edge state illustrated in the band structure [Fig. S8(a)].

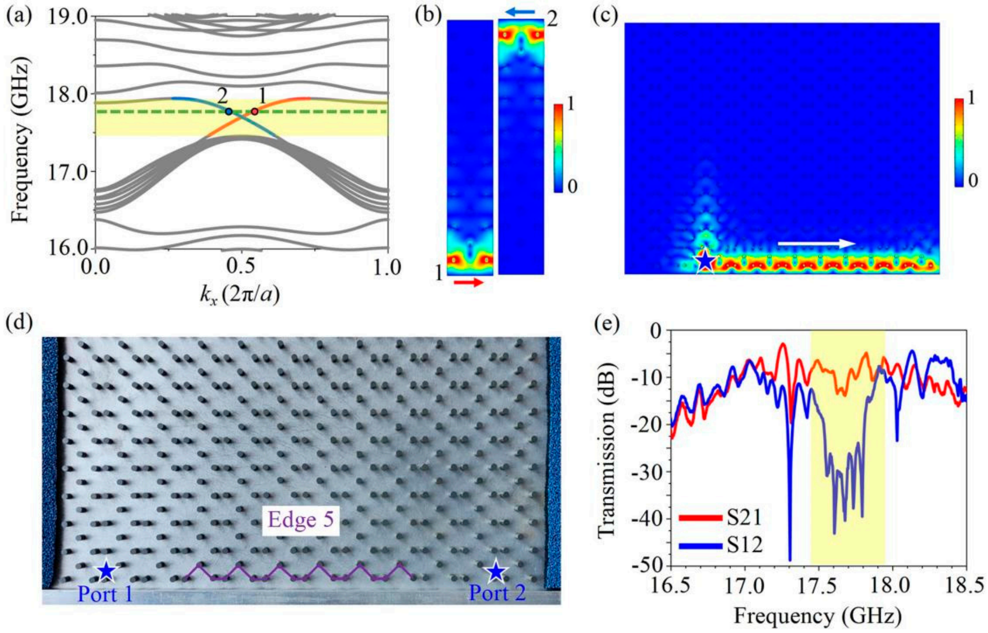

Figure S8. One-way edge states of Edge 5. (a) Projected band structures, showing two confined edge states within the photonic bandgap. (b) Eigenmodal fields of state 1 and state 2 denoted in (a). (c) Simulated electric field distribution excited by a line source illustrating one-way transport of edge state. (d) Photo picture of experimental sample. Edge 5 is highlighted in a purple polyline at the lower edge. (e) Measured transmission spectra showing excellent one-way transport properties of edge states.

V. Demonstration of the transport robustness of one-way edge states

In the above, we have presented systematical demonstrations and discussions on the one-way property of edge states that exist at five types of edges in the square-hexagon lattice GPC structure. Now, we proceed to theoretically and experimentally verify the transport robustness of these edge states in the presence of metallic obstacles on the path. To prove this, we insert a metallic obstacle of width 3.0 mm and height 20.0 mm in the lower edge. In simulations, when a line source (blue star) oscillating at f_s is laid at the lower edge, as seen in Figs. S9(a1)-S9(e1), energy fluxes can bypass the metallic obstacles with no back-reflections by creating a new one-way interface between the bulk of GPC and metallic obstacle. The corresponding experimental measurements are shown in Figs. S9(a2)-S9(e2) respectively. There are five regions with big contrast between S21 and S12 in the transmission spectra, in complete agreement with the theoretical results. Compared with the transmission spectra measured without the metallic obstacle, the forward transmission characteristics remain basically unchanged, despite the small changes in their amplitudes. Both simulation and measurement results show that these edge states are truly one-way edge states that are strongly robust against the backscattering and immune to the existence of metallic obstacles on the transport path.

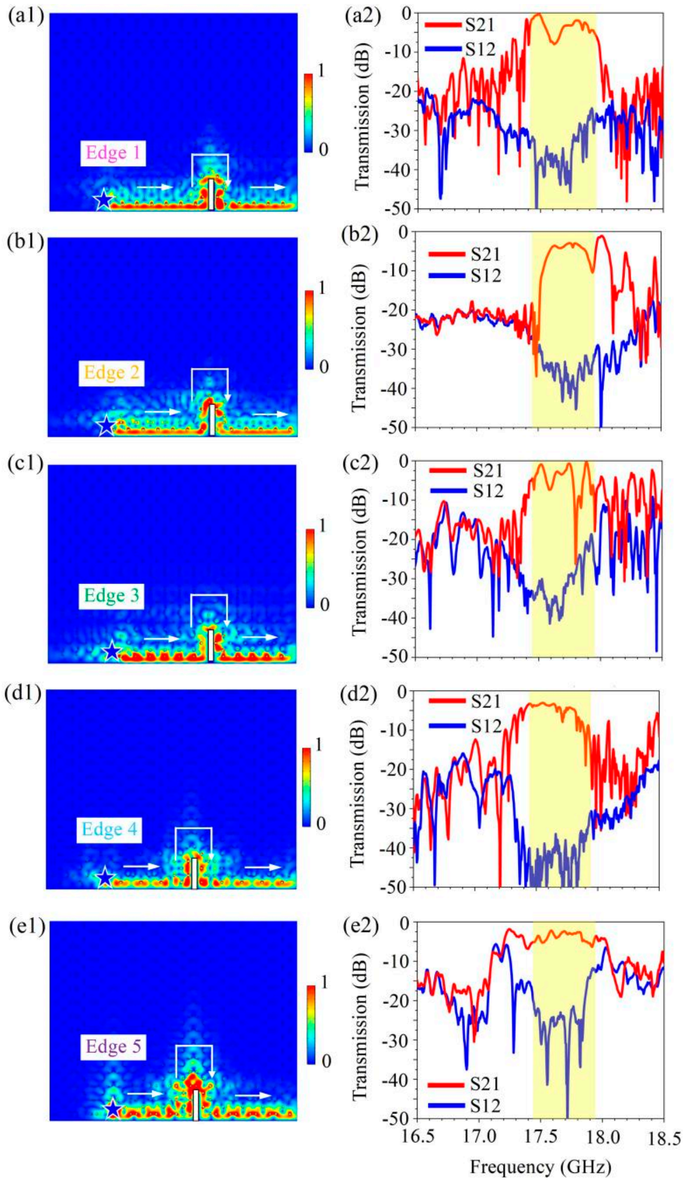

Figure S9. Transport robustness of one-way edge-states at five types of edges. (a1)-(e1) Simulated electric field distribution excited by a line source illustrating topologically one-way transport of edge state. (a2)-(e2) Measured transmission spectra showing excellent topologically protected one-way transport properties of edge states.
